# Medical student selection criteria as predictors of intended rural practice following graduation

**DOI:** 10.1186/1472-6920-14-218

**Published:** 2014-10-14

**Authors:** Ian B Puddey, Annette Mercer, Denese E Playford, Sue Pougnault, Geoffrey J Riley

**Affiliations:** Faculty Office, Faculty of Medicine, Dentistry and Health Sciences, The University of Western Australia, 35 Stirling Hwy, Crawley, WA 6009 Australia; School of Primary, Aboriginal and Rural Health Care, Faculty of Medicine, Dentistry and Health Sciences, The University of Western Australia, 35 Stirling Hwy, Crawley, WA 6009 Australia

## Abstract

**Background:**

Recruiting medical students from a rural background, together with offering them opportunities for prolonged immersion in rural clinical training environments, both lead to increased participation in the rural workforce after graduation. We have now assessed the extent to which medical students’ intentions to practice rurally may also be predicted by either medical school selection criteria and/or student socio-demographic profiles.

**Methods:**

The study cohort included 538 secondary school-leaver entrants to The University of Western Australia Medical School from 2006 to 2011. On entry they completed a questionnaire indicating intention for either urban or rural practice following graduation. Selection factors (standardised interview score, percentile score from the Undergraduate Medicine and Health Sciences Admission Test (UMAT) and prior academic performance (Australian Tertiary Admissions Rank), together with socio-demographic factors (age, gender, decile for the Index of Relative Socioeconomic Advantage and Disadvantage (IRSAD) and an index of rurality) were examined in relation to intended rural or urban destination of practice.

**Results:**

In multivariate logistic regression, students from a rural background had a nearly 8-fold increase in the odds of intention to practice rurally after graduation compared to those from urban backgrounds (OR 7.84, 95% CI 4.10, 14.99, P < 0.001). Those intending to be generalists rather than specialists had a more than 4-fold increase in the odds of intention to practice rurally (OR 4.36, 95% CI 1.69, 11.22, P < 0.001). After controlling for these 2 factors, those with rural intent had significantly lower academic entry scores (P = 0.002) and marginally lower interview scores (P = 0.045). UMAT percentile scores were no different. Those intending to work in a rural location were also more likely to be female (OR 1.93, 95% CI 1.08, 3.48, P = 0.027), to come from the lower eight IRSAD deciles (OR 2.52, 95% CI 1.47, 4.32, P = 0.001) and to come from Government vs independent schools (OR 2.02, 95% CI 1.15, 3.55, P = 0.015).

**Conclusions:**

Very high academic scores generally required for medical school entry may have the unintended consequence of selecting fewer graduates interested in a rural practice destination. Increased efforts to recruit students from lower socioeconomic backgrounds may be beneficial in terms of an ultimate intended rural practice destination.

**Electronic supplementary material:**

The online version of this article (doi:10.1186/1472-6920-14-218) contains supplementary material, which is available to authorized users.

## Background

In Australia and New Zealand, there have been two strategies implemented that have been clearly demonstrated to increase the likelihood of medical graduates opting for rural practice after graduation [1]. The first has been a major focus on recruiting students with rural backgrounds into medical schools [2–4]. The second has been to maximise opportunities for students to be imbedded in prolonged rural clinical clerkships [2]. The extent to which selection factors for medical school entry augment or inhibit the selection of students with an intended ultimate rural practice destination has to a large degree not been assessed. Studies of selection factors for entry into medical school have usually focused on academic performance throughout the course as the major criterion for assessing their predictive validity [5], rather than on any evaluation of potential links to ultimate career destination. Performance in selection tests is also influenced by socio-demographic factors [6, 7] and so any consideration of how selection factors might be associated with ultimate career destination also needs to take into account such potential confounding influences.

The selection process at The University of Western Australia (UWA) integrates an academic entry score - the Australian Tertiary Admissions Rank (ATAR), an interview score and the score from the Undergraduate Medicine and Health Sciences Admission Test (UMAT). The ATAR ranges between 99.95 and zero and is calculated on the basis of the total number of students in each annual examination cohort and the tertiary entrance aggregate distribution for that year [8]. The interview comprises a highly structured panel interview with a focus on communication skills. A comprehensive description of our interview process has been previously published [5]. The Undergraduate Medicine and Health Sciences Admission Test (UMAT) is comprised of 3 subtests which are developed each year by the Australian Council for Educational Research (ACER) on behalf of a group of Australian universities which form the UMAT Consortium [9]. The test is promoted as enhancing a focus on selection based on general attributes and non-academic personal skills gained through prior experience and learning and is designed to complement academic results used in selection processes. In subtest 1 (UMAT-1 - Logical reasoning and problem solving) candidates are required to exercise skills that utilise both inductive and deductive reasoning with an emphasis on logical argument in working to a solution. In subtest 2 (UMAT-2 - Understanding People) the emphasis is on assessing empathy and emotional intelligence with candidates required to show an understanding of the thoughts, feelings, behaviour and intentions portrayed within each question. Subtest 3 (UMAT-3 – Non-verbal Reasoning) is designed to obtain a measure of cognitive ability which is relatively independent of language ability or specific cultural knowledge. Following interview a composite score from the three criteria (ATAR, UMAT score and interview score) is utilised to produce a final ranked list from which offers are made.

Students can apply for admission to our medical school through a special entry pathway that quarantines places for rural background students. Such applicants generally have lower mean academic entry scores and lower total UMAT scores (unpublished data). Those subsequently selected for interview achieve similar scores at interview to their urban counterparts. Rural background entrants who are ultimately selected for entry generally have lower mean scores in all 3 selection criteria [7] and if places in the medical school were not quarantined for rural student entry these students would be at a significant disadvantage. The observation of a lower academic entry score in those from rural versus urban school environments has been a consistent outcome in previous Australian studies [10] and in a study of students from 6 Canadian medical schools a lower GPA on entry has also been linked to size of community of origin [11]. Whether these observations are due to the quality of rural secondary education, curriculum issues with fewer subject choices, lack of positive parental, teacher or peer encouragement or relative isolation with less face to face teaching is speculative. For Australian candidates for the UMAT, we have previously reported that performance in each of its 3 components can be differentially influenced by gender, ethnicity, age, Government vs independent school background, and relative socio-economic advantage or disadvantage [6]. The association of rural background with each UMAT component score is mixed with better performance in UMAT-1 and UMAT-2 but poorer performance in UMAT-3 [6]. These divergent associations with each of the 3 UMAT component scores results in total UMAT scores which are higher in those from areas that are defined as accessible or moderately accessible compared to those from highly accessible areas. These observations raise the possibility that if selection processes were modified to either lower the minimum academic entry scores required for entry and/or differentially weight the 3 sections of the UMAT, then selection of rural students into medical schools might be enhanced. The ultimate justification, however, for any changes to medical school selection criteria would need to be a convincing demonstration that it could lead to enhancement in the rural medical workforce without any compromise in the quality of graduates. In the current study, therefore we have analysed intended career destinations of commencing medical students against the criteria utilised for their selection into medical school, together with socio-demographic factors that could potentially influence rural versus urban practice intention.

## Methods

Those eligible for the analysis included all high school students who entered the standard admission pathway at The University of Western Australia from 2006 to 2011 and subsequently completed a question on preferred geographic region for ultimate practice destination (Table 1). The question was embedded in a questionnaire for the Medical Students Outcomes Database and Longitudinal Tracking Project that all commencing medical students in Australia and New Zealand are asked to complete [12]. Students indicated a preference for either a capital city, major urban centre, regional city or large town, a smaller town or a small community. This was subsequently grouped into a single dichotomous variable with the first 2 categories designated urban and the final 3 categories as rural. Students also completed a question on future career preference for generalist vs specialist practice. Those who indicated an interest in general practice, rural and remote medicine or indigenous health were classified as generalist. Indigenous, international and graduate entry students who were admitted through alternative selection pathways were excluded from the analysis.

**Table 1:** **Description of the University of Western Australia standard pathway entrants from 2006 to 2011**

	Year of entry to the course						
	2006	2007	2008	2009	2010	2011	Total
Standard pathway entrants	106	93	101	124	122	121	667
Ineligible for inclusion							
Did not complete MSOD questionnaire	24	4	9	12	12	17	78
Did not respond to question on intended practice location	7	15	10	7	3	9	51
Included in the study	75	74	82	105	107	95	538

Each of the three component scores, UMAT-1 (Logical reasoning and problem solving), UMAT-2 (Understanding people) and UMAT-3 (Non-verbal reasoning), have different and independent constructs and were therefore independently analysed in this study together with the total score. The UMAT scale, however, has changed over time and as a result scores are not necessarily comparable between years. Therefore percentile ranks which enable a measure of the relative standing of a candidate within each annual cohort have been utilised in this study rather than the raw score.

For urban background students the selection factors utilised for undergraduate entry at The University of Western Australia – ATAR, interview score and UMAT score - were weighted in a ratio of 1 : 1 : 1 to determine a final composite selection score. From 2008 onwards this was altered to a ratio of 2 : 2 : 1, respectively. In addition, rural students are selected into quarantined positions which have increased from 20% in 2006 to 29% of all standard entrants by 2011. The selection of rural students since 2007 has relied on one further factor – an Index of Rurality. This is generated from the Accessibility/Remoteness Index of Australia (ARIA) score [13] for the postal address of the secondary school (or secondary schools) attended by the student for each of the 5 years before application for selection and thus weights selection towards those rural students who completed their education at a rural secondary school. The selection factors for rural students are weighted in a ratio of 1 : 1.2 : 1.2 : 0.6 for Index of Rurality, ATAR, interview score and UMAT score, respectively.

Our definition of "rural" has evolved over time. In 2006 and 2007 applicants were considered rural if they had lived in a rural area of Western Australia for a minimum of two years and, during that period, completed year 12 at a rural secondary school – "rural" being defined as a distance of >75 kms from the Perth Central Business District. The rural definition was changed in 2008 in line with the Commonwealth of Australia Department of Health & Ageing definition to having a principal home address in a defined rural area of Australia (Rural, Remote and Metropolitan Areas 3–7) [14] for a minimum of 5 years (consecutive or cumulative) from the commencement of primary school. In 2010 the Commonwealth further refined the definition to having your principal home address in an Australian Standard Geographical Classification - Remoteness Area (ASGC-RA) [15] 2–5 for a minimum of 5 years (consecutive or cumulative) from the commencement of primary school.

As a socioeconomic indicator, the correspondence postcode at entry for each student was linked to the Index of Relative Socioeconomic Advantage and Disadvantage (IRSAD) score from the Australian 2006 census Socio-Economic Indices for Areas (SEIFA) [16]. The IRSAD score is derived by principal components analysis of 21 separate variables such as low or high income, internet connection, unemployment, occupation and education. The score is standardised against a mean of 1000 with a standard deviation of 100. The IRSAD decile score was utilised in regression because SEIFA codes are not linear. A dummy variable was constructed which dichotomised the cohort into the top 2 deciles vs the bottom 8 deciles because two thirds of the study population were within the top 2 deciles with increasingly smaller numbers across the other 8 deciles.

The academic performance of the final selected cohort was assessed from the weighted average mark for the year for levels 1 through 6 of the course. The Weighted Average Mark (WAM) calculated for each of these levels used results (expressed as percentages) for core units weighted by the UWA points system for the size of the unit.

This study is part of a larger ongoing project which includes all school-leaver entrants who undertake the full 6-year course at The University of Western Australia. Results of that part of the project have been reported elsewhere [5]. The project has been approved by the Human Research Ethics Committee at UWA as an amendment to the larger project (file reference RA/4/1/2178). The MSOD Research and Scientific Advisory Committee also provided approval for utilisation of the MSOD questionnaire data and all MSOD participants provided informed written consent.

### Statistical analysis

Data were analysed using IBM SPSS Statistics Release 20.0.0. All values are reported as Mean ± SEM. Univariate comparisons by rural vs urban background were made using the χ^2^ test for categorical variables and unpaired T-test for each selection factor. Univariate analysis of intention to practice in a rural vs urban environment was analysed by logistic regression for categorical variables and unpaired T-test for each selection factor. Multivariate logistic regression models were then constructed for the major outcome variable of ultimate intended destination of practice after graduation, using the full set of selection factors together with the socio-demographic variables outlined above as predictor variables. The models included adjustment for previously demonstrated associations of rural background and generalist vs specialist career intention on ultimate intended practice destination.

## Results

### Summary statistics

There were 667 subjects eligible for the analysis, of whom 78 did not complete the MSOD questionnaire at entry to medical school and 51 did not respond to the question on intended practice location (Final effective sample size, N = 538, completion rate 81%). A comparison of the selection scores (UMAT and each of its components, academic entry score and interview score) in those who completed the questionnaire versus those who did not, revealed no significant differences in academic entry score, total UMAT percentile score or the score in UMAT-1 and UMAT-2. However, those who did not complete either the questionnaire or the specific question on preferred geographic region for ultimate practice destination, did have a significantly lower UMAT-3 percentile score (75.3 ± 1.8 vs 80.3 ± 0.8, Unpaired T-test, P = 0.008) and a higher interview score (27.8 ± 0.5 vs 26.7 ± 0.2, Unpaired T-test, P = 0.031). There were no significant differences in the distribution for both groups in terms of age, gender, rural background, IRSAD decile and type of high school attended.

The final cohort had a mean age of 18.3 ± 0.03 yr at entry to the course. Approximately 75% were of urban origin (N = 404) and 25% of rural origin (N = 134); 55% were female (N = 294) and 45% were male (N = 244); 70% were from an independent (fee paying) school background (N = 371) and 30% from a Government school background (N = 159); 32% were from the lower 8 IRSAD deciles (N = 174) and 68% from the upper 2 deciles (N = 364). This profile was not significantly different for each year of the cohort from 2006 to 2011.

Selection criteria and socio-demographic factors by rural vs urban background are listed in Table 2. Rural students had lower academic entry scores, interview scores and UMAT percentile scores on entry. Rural students were older and more likely to come from an area of reduced socio-economic advantage and increased socioeconomic disadvantage. There was no significant gender difference and no difference in Government vs independent secondary school background.

**Table 2:** **Selection criteria**, **socio**-**demographic factors and intended specialty by rural vs urban background**

Variable	N	Urban background	N	Rural background	P-Value (χ ^2^ test)
Academic entry score (ATAR)	404	99.1 ± 0.04	134	97.9 ± 0.11	<**0.001**
Interview Score	404	27.0 ± 0.3	134	25.5 ± 0. 4	**0.004**
Total UMAT percentile score	404	89.8 ± 0.4	134	76.2 ± 1.3	<**0.001**
UMAT-1 percentile score	404	86.0 ± 0.6	134	77.3 ± 1.6	<**0.001**
UMAT-2 percentile score	404	78.3 ± 1.0	134	74.0 ± 1.6	**0.028**
UMAT-3 percentile score	404	85.0 ± 0.7	134	66.1 ± 1.9	<**0.001**
Age on admission					**0.001**
Up to 18 yr	314	77.7%	76	56.7%	
19 yr or older	90	22.3%	58	43.3%	
Sex					NS
Male	183	45%	61	45%	
Female	221	55%	73	55%	
Secondary school					NS
Independent	281	71%	90	67%	
Government	115	29%	44	33%	
IRSAD score					<**0.001**
Deciles 1-2	1	0.2%	3	2.2%	
Deciles 3-4	6	1.5%	7	5.2%	
Deciles 5-6	24	5.9%	43	32.1%	
Deciles 7-8	63	15.6%	27	20.1%	
Deciles 9-10	310	76.7%	54	40.3%	
ASGC-RA					<**0.001**
Major cities	401	99.3%	2	1.5%	
Inner regional	3	0.7%	52	38.8%	
Outer regional	0	0	48	35.8%	
Remote	0	0	30	22.4%	
Very remote	0	0	2	1.5%	
Intended specialty					<**0.001**
Undecided	169	42.1%	49	37.1%	
Specialist	216	53.9%	59	43.7%	
Generalist	16	4%	24	18.2%	

Significant P values are in bold-faced type.

### Univariate analysis of the selection criteria

Given that the rural students were recruited with lower overall academic entry scores, interview scores and UMAT percentile scores, the univariate analysis for each group was conducted separately. Comparisons of each selection factor vs intended rural vs urban destination are outlined in Table 3 for urban background subjects and Table 4 for those from a rural background. There was no difference in the total UMAT percentile score, scores in each of the UMAT components or interview scores in those intending to work in a rural vs urban location. The academic entry score was lower in urban background students who intended to practice rurally with a similar (but non-significant) trend seen in rural background students with both a lower academic entry score and lower UMAT-3 score in those intending to practice rurally.

**Table 3:** **Selection factors by intended urban vs rural site of practice in urban background subjects**

	Intended site of practice	N	Mean ± SEM	P value (Unpaired T-Test)
Total UMAT percentile score	Urban	365	89.9 ± 0.4	
	Rural	39	88.3 ± 1.4	0.228
UMAT-1 percentile score	Urban	365	86.2 ± 0.7	
	Rural	39	84.7 ± 2.2	0.499
UMAT-2 percentile score	Urban	365	78.4 ± 1.0	
	Rural	39	76.8 ± 2.8	0.616
UMAT-3 percentile score	Urban	365	84.9 ± 0.8	
	Rural	39	85.8 ± 2.0	0.701
Interview score	Urban	365	27.2 ± 0.27	
	Rural	39	25.9 ± 0.82	0.135
Academic entry score (ATAR)	Urban	365	99.2 ± 0.04	
	Rural	39	98.7 ± 0.13	**0.001**

Significant P values are in bold-faced type.

**Table 4:** **Selection factors by intended urban vs rural site of practice in rural background subjects**

	Intended site of practice	N	Mean ± SEM	P value (Unpaired T-Test)
Total UMAT percentile score	Urban	47	77.0 ± 2.4	
	Rural	87	75.8 ± 1.6	0.659
UMAT-1 percentile score	Urban	47	78.2 ± 2.5	
	Rural	87	76.8 ± 2.0	0.666
UMAT-2 percentile score	Urban	47	72.3 ± 3.1	
	Rural	87	74.9 ± 1.9	0.451
UMAT-3 percentile score	Urban	47	70.4 ± 3.0	
	Rural	87	63.8 ± 2.4	0.101
Interview score	Urban	47	25.3 ± 0.27	
	Rural	87	25.7 ± 0.82	0.700
Academic entry score (ATAR)	Urban	47	98.2 ± 0.18	
	Rural	87	97.8 ± 0.13	0.078

### Univariate analysis of the socio-demographic data

The univariate analysis of socio-demographic predictors of intention of students to work in a rural environment are outlined in Table 5. Rural background was the strongest predictor with a more than 17-fold increase in the odds of intending to practice rurally. For rural background students alone, the Index of Rurality used as an additional selection factor did not predict an increasing likelihood of an intention to practice rurally (data not shown). Similarly for all subjects, increasing rurality as measured by ASGC was not associated with a commensurate increased likelihood of working rurally (Table 5). There was however, a significant and increasing trend for those from the lower 8 IRSAD deciles to indicate an intention to work rurally compared to those in the upper 2 deciles. Students from Government schools had a small but significant increase in the odds of rural practice intention.

**Table 5:** **Univariate predictors for intention of working in a rural environment**

	Number (%) intending rural site of practice	Odds ratio (Logistic regression)	*P*
Rural background			
Yes	87/134 (64.9%)	17.32 (10.67, 28.13)	<**0.001**
No	39/404 (9.7%)	1.0	
Intended specialty			
Undecided	39/218 (17.9%)	1.0	
Specialist	60/275 (21.8%)	1.28 (0.82, 2.01)	0.280
Generalist	25/40 (62.5%)	7.65 (3.69, 15.84)	<**0.001**
Sex			
Male	49/244 (20.1%)	1.0	
Female	77/294 (26.2%)	1.41 (0.94, 2.12)	0.097
Age on admission			
Up to 18 yr	72/390 (18.5%)	1.0	
19 yr	54/148 (36.5%)	2.54 (1.67, 3.87)	<**0.001**
ASGC			
Major cities	40/403 (9.9%)	1.0	
Inner regional	38/55 (69.1%)	20.29 (10.50, 39.19)	<**0.001**
Outer regional	28/48 (58.3%)	12.71 (6.57, 24.59)	<**0.001**
Remote and Very remote	20/32 (62.5%)	15.13 (6.89, 33.22)	<**0.001**
IRSAD score			
Deciles 1-2	3/4 (75%)	17.60 (1.80, 172.42)	**0.014**
Deciles 3-4	6/13 (46.2%)	5.03 (1.63, 15.55)	**0.005**
Deciles 5-6	33/67 (49.3%)	5.70 (3.25, 9.98)	<**0.001**
Deciles 7-8	31/90 (34.4%)	3.08 (1.83, 5.20)	<**0.001**
Deciles 9-10	53/364 (14.6%)	1.0	
Secondary school			
Independent	77/371 (20.8%)	1.0	
Government	48/159 (30.2%)	1.65 (1.08, 2.52)	**0.020**
RCS application			
Did not apply for place in the RCS	24/223 (10.8%)	1.0	
Applied for place in the RCS	58/184 (31.5%)	3.82 (2.26, 6.46)	<**0.001**

Significant P values are in bold-faced type.

In the cohort there were 407 students who had either completed or entered level 5 of the MBBS program and therefore had the opportunity to be selected for a full year placement in our Rural Clinical School. Students who applied for a place in the RCS exhibited a nearly 4-fold increase in the odds of preferring a rural vs urban ultimate destination of practice than those who did not apply to the RCS (OR 3.82, 95% CI 2.26, 6.46, P < 0.001) (Table 5).

### Multivariate analyses of selection factors and socio-demographic data

The final multivariate logistic regression model is outlined in Table 6. Students from a rural background had a nearly 8-fold increase in the odds of indicating an intent to practice rurally after graduation compared to those from urban backgrounds (OR 7.84, 95% CI 4.10, 14.99, P < 0.001). Those intending to practice as generalists were more than 4-fold more likely to intend to practice rurally (OR 4.36, 95% CI 1.69, 11.22). After controlling for these 2 factors, those intending to practice rurally were predicted by significantly lower academic entry scores (P = 0.002) on selection to the course and marginally lower interview scores (P = 0.045). No component of the UMAT predicted rural career intention. Those indicating an intention to work in a rural location on graduation were more likely to be female (OR 1.93, 95% CI 1.08, 3.48, P = 0.027), to come from the lower eight IRSAD deciles (OR 2.52, 95% CI 1.47, 4.32, P = 0.001) and to come from Government vs independent secondary schools (OR 2.02, 95% CI 1.15, 3.55, P = 0.015).

**Table 6:** **Multivariate logistic regression with intended urban vs rural site of practice as the dependent variable and rural background**, **selection factors**, **intended specialty and socio**-**demographic variables as the predictor variables** (**N** = **525**) (**Nagelkerke R Square** = **0.473**)

Predictor variable	B value	S.E. for B	P value	Odds ratio	95% CI for odds ratio	
					Lower	Upper
Rural background						
Urban				1		
Rural	2.059	0.331	<**0.001**	7.84	4.10	14.99
Intended specialty						
Undecided				1		
Specialist	0.426	0.291	0.143	1.53	0.87	2.71
Generalist	1.472	0.483	**0.002**	4.36	1.69	11.22
Age						
18 yr or younger				1		
19 yr or older	0.430	0.285	0.132	1.54	0.88	2.69
Sex						
Male				1		
Female	0.660	0.299	**0.027**	1.93	1.08	3.48
IRSAD score						
Deciles 9-10				1		
Deciles 1-8	0.923	0.276	**0.001**	2.52	1.47	4.32
School type						
Independent				1		
Government	0.702	0.288	**0.015**	2.02	1.15	3.55
Academic entry score (ATAR)	-0.430	0.140	**0.002**	0.65	0.50	0.86
Interview score	-0.054	0.027	**0.045**	0.95	0.90	1.00
UMAT-1 percentile score	0.007	0.009	0.487	1.01	0.99	1.03
UMAT-2 percentile score	-0.004	0.007	0.619	1.00	0.98	1.01
UMAT-3 percentile score	0.000	0.008	0.978	1.00	0.99	1.02

Significant P values are in bold-faced type.

Multivariate logistic regression models were also constructed for urban and rural background subjects separately (Additional file 1: Tables S1 and S2). For urban background students, a generalist practice intention, being from lower IRSAD deciles, and both lower academic entry scores and interview scores remained predictive of intention to practice rurally. For rural background students, being from lower IRSAD deciles and from Government vs independent secondary schools remained as independent predictors.

### Academic performance

The Weighted Average Mark achieved each year was used to monitor the academic performance of each student as they progressed through the course. The results for those who expressed a rural vs urban preference for ultimate career destination are outlined in Table 7. The WAM was significantly lower in those indicating a rural preference from levels 1 through 4. However, this was no longer the case at exit from the course with no significant difference apparent at levels 5 and 6. To some extent this may have been explained by a greater proportion of subjects among those expressing a rural preference subsequently withdrawing from the course (12.7% of rural students vs 3.9% of urban students, P < 0.001) or being withdrawn because of unsatisfactory academic progression (10.3% of rural students vs 1.9% of urban students, P < 0.001) (Table 8). The independent predictors of rural vs urban intended destination of practice were unchanged in a final multivariate model which excluded all subjects who subsequently withdrew from the course.

**Table 7:** **Academic performance during the course by intended site of practice**

	Intended site of practice	N	Weighted average mark (Mean ± SEM)	P value (Unpaired T-Test)
Year 1	Urban	410	72.9 ± 0.4	
	Rural	125	68.3 ± 0.8	<**0.001**
Year 2	Urban	403	70.3 ± 0.4	
	Rural	119	65.7 ± 0.8	<**0.001**
Year 3	Urban	397	72.8 ± 0.4	
	Rural	111	69.4 ± 0.7	<**0.001**
Year 4	Urban	323	72.2 ± 0.3	
	Rural	79	69.4 ± 0.7	**0.002**
Year 5	Urban	237	74.1 ± 0.3	
	Rural	51	73.4 ± 0.6	0.280
Year 6	Urban	157	73.1 ± 0.3	
	Rural	33	72.4 ± 0.8	0.335

Significant P values are in bold-faced type.

**Table 8:** **Withdrawal from the course by year of entry**

Year of entry to the course									
		2006	2007	2008	2009	2010	2011	Total	χ ^2^ (P-value)
Withdrawn (any reason)	Urban	3/54 (5.6%)	5/61 (8.2%)	3/71 (4.2%)	1/81 (1.2%)	1/77 (1.3%)	3/68 (4.4%)	16/412 (3.9%)	13.43 (<**0.001**)
	Rural	5/21 (28%)	3/13 (23.1%)	1/11 (9.1%)	1/24 (4.2%)	2/30 (6.7%)	4/27 (14.8%)	16/126 (12.7%)	
Withdrawn (unsatisfactory academic progression)	Urban	2/54 (3.7%)	1/61 (1.6%)	1/71 (1.4%)	1/81 (1.2%)	1/77 (1.3%)	2/68 (2.9%)	8/412 (1.9%)	18.05 (<**0.001**)
	Rural	4/21 (19%)	3/13 (23.1%)	0/11 (0%)	0/24 (0%)	2/30 (6.7%)	4/27 (14.8%)	13/126 (10.3%)	

Significant P values are in bold-faced type.

## Discussion

The Medical Schools Outcomes Database and Longitudinal Tracking Project commenced in 2005 and tracks all Australian medical students from their entry into medical school and into the postgraduate environment [17]. Data from the entry questionnaires for the first 3 cohorts from 2005 to 2007 have already been reported in relation to factors predictive of a student’s intention to practice rurally after graduation [17, 18] with the strongest predictors being an intention to generalist rather than specialist practice, older age and coming from a rural residential background. This rural background effect is now well documented in Australia [19] and internationally [20–23] with a systematic review of 12 studies concluding that rural background increased the odds of rural practice 2 to 2.5 fold [24]. In the current study, which evaluated intentions rather than actual rural practice, the odds were much higher, with rural background students nearly 8 times more likely to indicate an ultimate rural site of practice. The current study has also been able to further evaluate the extent to which selection factors utilised for medical school along with socio-demographic profile might also influence students’ intentions to practice rurally, even after taking this established strong rural background effect into account. We have observed that those intending to practice rurally had significantly lower academic entry scores on admission together with marginally lower interview scores while UMAT scores were not significantly different. We have also observed that intention to practice rurally was associated with being female, being older, being from the lower socio-economic deciles, being from a Government vs independent school background and having an intention for generalist vs specialist practice.

The trend for lower academic entry scores in those who indicated an intention to practice rurally was evident in both urban and rural background students. We have previously reported that the most powerful predictor of performance throughout our MBBS course for both undergraduate [5] and graduate entrants [25] is prior academic performance as assessed by either ATAR or tertiary grade point average, respectively. It was therefore reassuring that those indicating at entry to the course an intention to practice rurally were performing at a similar level academically at exit from the course. This may be indicative that any move to lower the threshold ATAR for entry as an approach to increasing the likelihood of students practicing rurally will not necessarily result in a compromise in the ultimate quality of our medical school graduates. However, this would need to be balanced against the evidence in this study that withdrawal due to subsequent unsatisfactory academic progression was greater in those who indicated a preference for rural vs urban destination of practice.

UMAT scores in this study were not a significant predictor of rural practice intent. This observation provides no support for consideration of differential weighting of the component parts of the UMAT in the selection of medical students as a way to augment recruitment of students more likely to practice rurally. Students from the lower socio-economic deciles, those from Government vs independent schools, those from rural areas, older students and females all exhibit lower total UMAT scores when first sitting the test [6]. However the scores on the different components may be affected contrarily. For example, in subtest 2 section - Understanding People – females, older students and rural students score better while in subtest 3 - Non-verbal Reasoning – these same 3 groups all exhibit lower scores [6]. Differential weighting of component scores as a means to increase the competitiveness of rural background students could therefore have broader consequences in terms of the ultimate effects on the socio-demographic profile of the student cohort selected, rather than just serve to augment rural student selection alone. An approach to quarantine places within a medical school for rural students who reach acceptable thresholds for total UMAT score probably therefore represents the best alternative in attempts to maximise rural student recruitment.

In a broader context, the use of aptitude tests internationally for medical selection has been driven in part by the anticipation that this would widen access into medical schools. However, use of the UMAT in Australia and New Zealand [6] and use of the MCAT in Canada [11] have suggested lower performance outcomes in those with socio-economic disadvantage. In the United Kingdom, utilisation of the UKCAT has had mixed outcomes in this regard. James et al. [26] found that white ethnicity, having parents from a professional/managerial background and independent or grammar schooling each were independent indicators of more favourable UKCAT performance. In contrast, Tiffin et al. [27] found variable results with either favourable or unfavourable outcomes in widening access depending on the way the UKCAT score was ultimately applied in the selection of candidates for subsequent interview and an offer of a place. There is therefore, limited evidence that the use of aptitude tests can actually widen access into medical schools. In the recruitment of rural background students this provides further support for an approach that quarantines places for applicants who reach pre-requisite thresholds when aptitude tests are utilised in selection.

We have recently evaluated the effect of rural background on a cohort of students admitted to our medical school before the MSOD tracking project began but where actual site of practice could be determined a minimum 3 years after graduation. In that study a rural background was predictive of a 4-fold increase in the odds of practicing rurally [1]. The students in the current cohort, however, either are still progressing through the course or in the early stages of postgraduate training with only those who commenced in 2006 now entering their third postgraduate year. Hence further follow-up will be necessary to assess the extent to which stated intent translates into actual rural practice. However, the observation that the students expressing an intention to practice rurally at entry to the course were approximately 4-fold fold more likely to apply for a year-long rotation in our Rural Clinical School at level 5 of the course is an encouraging sign that intent may indeed translate into an actual rural career for a substantial number [1]. Results from the Jefferson Longitudinal Study of Medical Education also support the durability of an indication of initial intent for rural practice at entry to medical school as a predictor for subsequent actual practice in a rural area [28]. However, a study limited to analysis of results from a questionnaire at entry to medical school is unable to take into account the subsequent influence (either negative or positive) of a medical school environment to change initial career intentions. In this regard in a recent study of 4 Scottish medical schools [29], where career intentions were assessed both at entrance and exit from medical school, there were not only socio-demographic differences in each student cohort that dictated ultimate career choices, but also relevant institution-specific differences in medical school education and culture that were influencing outcomes. In our own medical school the presence of a Rural Clinical School, specific rural student support structures, rural-interest student clubs and short term general practice attachments in rural locations may all be operating to change motivations with respect to an ultimate choice for rural versus urban practice.

Female students were twice as likely as males to indicate an intention to practice rurally. This contrasts with observations of female general practitioners and specialists in Australia being half as likely to be practicing in a rural location compared with males [19]. Barriers to practicing rurally are much greater for women than men with issues related to the high demands of rural practice relative to preferred part time practice in those with family life responsibilities [30]. Again, long term follow up of the women in our cohort will establish whether an expressed intent to practice rurally actually translates into ultimate rural practice.

A large number of the students in this cohort were, not surprisingly, uncertain of their ultimate career at entry to medical school. However, those who indicated interest in a generalist career were, like those in the larger MSOD cohort [18], more likely to indicate an intent to practice rurally. A similar observation has been reported for New Zealand medical students participating in the University of Auckland Tracking Health Professional Students and Graduates Project [31]. Of interest that project identified lower UMAT scores in all 3 components for those who indicated a strong interest in general practice at exit from the course [32], but with no identifiable differences in interview scores or grade point average from prior university achievement. The authors speculated that high cut points for medical school selection with the UMAT may exclude applicants with a propensity for general practice. They did not report as to whether any of their selection factors were also linked to an inclination towards rural practice.

Among the rural background students, neither the Index of Rurality used as an additional factor in their selection, nor coming from a town with a higher ASGC score, further enhanced the likelihood of students intending to practice rurally. In a similar fashion, analysis of New Zealand medical students has not shown any significant link between the size of the rural community from which students are recruited and a generalist vs specialist career intention [33]. This might bring into question the utilisation of our Index of Rurality as an additional selection factor for students from a rural background. However, given that it is generated on the basis of higher scores for those who attended secondary schools in rural areas, it serves to at least in part, redress the imbalance in terms of lower academic entry scores and UMAT scores that are seen in those from relatively disadvantaged rural backgrounds.

Students from the lower socio-economic deciles were 2.5 fold more likely to express an intention to practice rurally on graduation. Similarly those from Government rather than independent (fee-paying) secondary schools were twice as likely to express an intention to practice rurally. These associations were still seen even after allowing for rural background which in itself is associated with a lower SEIFA score [34]. Socio-economic factors are themselves important dictates of the current divide between physician supply to urban vs rural locations and so these observations may have implications into the future for a greater focus for medical school recruitment on students from disadvantaged socio-economic backgrounds. However, in a broader study of all MSOD participants between 2005 and 2009, where Jones et al. [34] utilised the postcode of the secondary school attended to generate a SEIFA code for each participant rather than their correspondence address, it was reported that those who attended schools with the lowest socio-economic scores had a decrease in the odds of indicating a rural practice intention. It is difficult to reconcile this result with our data. However, they used the Index of Relative Socio-economic Disadvantage score (IRSD) rather than the IRSAD score. Higher scores in the IRSAD have more spread than the IRSD and indicate relative advantage as well as disadvantage, so that schools with a high disadvantage score will not necessarily also have a high advantage/disadvantage score. Although not stated they also appear to have used the IRSD score rather than IRSD decile in their regression analysis which as discussed earlier may violate the linearity assumption.

### Limitations of the study

The study was confined to a single medical school and the results may not be generalizable to other Australian medical schools or international institutions. The study relied only on a questionnaire at entry to medical school and not at exit. Rural intentions may therefore have been modified during the period in medical school. Moreover, student responses about rural practice intent so early in the course could have been influenced by social desirability bias, or suggestibility, especially given that up to a quarter had been selected on the basis of their rurality. The relatively small sample size may have introduced some bias in terms of non-responders and it was interesting in this regard that those who did not complete either the MSOD questionnaire or the specific question on preferred geographic region for ultimate practice destination, exhibited similar academic entry scores but lower UMAT-3 percentile scores and higher interview scores. During the 2006 to 2011 study recruitment period the definition of rural background has not been constant. However, it has always had as its major criterion the duration of time with a principal home address in a rural locality. The use of an individual’s postcode as a surrogate for socio-economic status imputes an index (IRSAD) for all people living in a defined area and may not be truly reflective of socio-economic status for each individual in that area [16].

## Conclusion

Without the quarantining of specific places for rural background students in a medical school the current emphasis on UMAT and academic entry scores for selection potentially places rural applicants in Australia at a disadvantage. The results of the current study suggest that the very high academic entry scores generally required for undergraduate medical school entry from secondary school may have the further unintended consequence of selecting fewer urban graduates interested in rural practice on graduation. They also indicate that increased efforts to recruit both urban and rural background students from areas of lower socio-economic advantage may bear fruit in terms of a greater likelihood of an ultimate intent to work rurally after graduation.

## Electronic supplementary material

Additional file 1: Table S1: Multivariate logistic regression with intended urban vs rural site of practice as the dependent variable and selection factors, intended specialty and socio-demographic variables as the predictor variables, for urban background students only (N=393) (Nagelkerke R Square = 0.183). **Table S2.** Multivariate logistic regression with intended urban vs rural site of practice as the dependent variable and selection factors, intended specialty and socio-demographic variables as the predictor variables, for rural background students only (N=132) (Nagelkerke R Square = 0.219). (DOCX 24 KB)
