# Supplementary material for: Medical student selection criteria as predictors of intended rural practice following graduation
Source: BMC Med Educ. 2014 Oct 14;14:218. doi: 10.1186/1472-6920-14-218 (PMC4287212; doi:10.1186/1472-6920-14-218)
Supplement: Supplementary file 1 — Additional file 1: Table S1: Multivariate logistic regression with intended urban vs rural site of practice as the dependent variable and selection factors, intended specialty and socio-demographic variables as the predictor variables, for urban background students only (N=393) (Nagelkerke R Square = 0.183). Table S2. Multivariate logistic regression with intended urban vs rural site of practice as the dependent variable and selection factors, intended specialty and socio-demographic variables as the predictor variables, for rural background students only (N=132) (Nagelkerke R Square = 0.219). (DOCX 24 KB) [file 12909_2014_1045_MOESM1_ESM.docx]

**Additional Table 1** - Multivariate logistic regression with intended urban vs rural site of practice as the dependent variable and selection factors, intended specialty and socio-demographic variables as the predictor variables, for urban background students only (N=393) (Nagelkerke R Square = 0.183).

| Predictor Variable | B value | S.E. for B | P Value | Odds Ratio | 95% CI for Odds Ratio | |
| --- | --- | --- | --- | --- | --- | --- |
|  |  |  |  |  | Lower | Upper |
| Intended specialty |  |  |  |  |  |  |
| Undecided |  |  |  | 1 |  |  |
| Specialist | 0.715 | 0.424 | 0.092 | 2.044 | 0.890 | 4.692 |
| Generalist | 2.016 | 0.687 | **0.003** | 7.505 | 1.952 | 28.847 |
| Age |  |  |  |  |  |  |
| 18 yr or younger |  |  |  | 1 |  |  |
| Age 19 yr or older | 0.117 | 0.426 | 0.783 | 1.124 | 0.488 | 2.592 |
| Sex |  |  |  |  |  |  |
| Male |  |  |  | 1 |  |  |
| Female | 0.512 | 0.411 | 0.213 | 1.669 | 0.745 | 3.737 |
| IRSAD Decile |  |  |  |  |  |  |
| 9-10 |  |  |  | 1 |  |  |
| 1-8 | 1.021 | 0.381 | **0.007** | 2.776 | 1.317 | 5.853 |
| School Type |  |  |  |  |  |  |
| Independent |  |  |  | 1 |  |  |
| Government | 0.345 | 0.390 | 0.377 | 1.412 | 0.657 | 3.036 |
| ATAR | -0.786 | 0.221 | **<0.001** | .456 | 0.295 | 0.703 |
| Interview Score | -0.103 | 0.039 | **0.009** | 0.902 | 0.835 | 0.975 |
| UMAT-1 Percentile Score | -0.005 | 0.014 | 0.733 | 0.995 | 0.968 | 1.023 |
| UMAT-2 Percentile Score | -0.007 | 0.010 | 0.477 | 0.993 | 0.974 | 1.013 |
| UMAT-3 Percentile Score | 0.005 | 0.014 | 0.718 | 1.005 | 0.978 | 1.032 |

Significant P values are in bold-faced type

**Additional Table 2** - Multivariate logistic regression with intended urban vs rural site of practice as the dependent variable and selection factors, intended specialty and socio-demographic variables as the predictor variables, for rural background students only (N=132) (Nagelkerke R Square = 0.219).

| Predictor Variable | B value | S.E. for B | P Value | Odds Ratio | 95% CI for Odds Ratio | |
| --- | --- | --- | --- | --- | --- | --- |
|  |  |  |  |  | Lower | Upper |
| Intended specialty |  |  |  |  |  |  |
| Undecided |  |  |  | 1 |  |  |
| Specialist | 0.107 | 0.435 | 0.807 | 1.113 | 0.474 | 2.611 |
| Generalist | 1.019 | 0.668 | 0.127 | 2.769 | 0.748 | 10.257 |
| Age |  |  |  |  |  |  |
| 18 yr or younger |  |  |  | 1 |  |  |
| Age 19 yr or older | 0.610 | 0.424 | 0.150 | 1.840 | 0.802 | 4.225 |
| Sex |  |  |  |  |  |  |
| Male |  |  |  | 1 |  |  |
| Female | 0.853 | 0.452 | 0.059 | 2.348 | 0.968 | 5.693 |
| IRSAD Decile |  |  |  |  |  |  |
| 9-10 |  |  |  | 1 |  |  |
| 1-8 | 0.870 | 0.420 | **0.038** | 2.387 | 1.048 | 5.438 |
| School Type |  |  |  |  |  |  |
| Independent |  |  |  | 1 |  |  |
| Government | 1.136 | 0.493 | **0.021** | 3.113 | 1.185 | 8.177 |
| ATAR | -0.267 | 0.185 | 0.149 | 0.766 | 0.533 | 1.101 |
| Interview Score | -0.032 | 0.042 | 0.447 | 0.969 | 0.893 | 1.051 |
| UMAT-1 Score | 0.010 | 0.013 | 0.439 | 1.010 | 0.985 | 1.036 |
| UMAT-2 Score | 0.001 | 0.011 | 0.929 | 1.001 | 0.979 | 1.023 |
| UMAT-3 Score | -0.004 | 0.010 | 0.706 | 0.996 | 0.977 | 1.016 |

Significant P values are in bold-faced type
